# The “Retrofitting” Approach to Adapting Evidence-Based Interventions: A Case Study of Pediatric Asthma Care Coordination, United States, 2010–2014

**DOI:** 10.5888/pcd13.160129

**Published:** 2016-08-25

**Authors:** Mary R. Janevic, Shelley C. Stoll, Marielena Lara, Gilberto Ramos-Valencia, Tyra Bryant Stephens, Victoria Persky, Kimberly Uyeda, Julie Kennedy Lesch, Floyd J. Malveaux

**Affiliations:** Author Affiliations: Shelley C. Stoll, University of Michigan School of Public Health, Ann Arbor, Michigan; Marielena Lara, RAND Corporation, Santa Monica, California; Gilberto Ramos-Valencia, University of Puerto Rico School of Public Health, San Juan, Puerto Rico; Tyra Bryant Stephens, Children’s Hospital of Philadelphia, Philadelphia, Pennsylvania; Victoria Persky, University of Illinois at Chicago School of Public Health, Chicago, Illinois; Kimberly Uyeda, Los Angeles Unified School District, Los Angeles, California; Julie Kennedy Lesch, Floyd J. Malveaux, Merck Childhood Asthma Network, Washington, DC.

## Abstract

Adaptation of evidence-based interventions upon implementation into new practice settings is universal, yet poorly understood. During a cross-site evaluation of the implementation of a proven intervention for pediatric asthma care coordination into 4 resource-challenged settings, we conducted in-depth interviews with site representatives, who reported how and why they modified intervention components. Interview notes were coded for themes. We focused on a single theme from a respondent who described the adaptation process as “backing” the intervention into ongoing services; we found evidence of a similar process at other sites. We labeled this process “retrofitting” to signify adaptation that consists of altering existing services to align with intervention components, rather than modifying the intervention to fit a new setting. Advantages of retrofitting may include allowing organizations to keep what works, capitalizing on existing support for program activities, elevating the role of local knowledge, and potentially promoting the sustainability of effective innovations.

## Background

Practitioners are increasingly being called on to use evidence-based approaches (1), often as a prerequisite for federal or health department funding (1,2). The need to modify these evidence-based interventions (EBIs) when they are implemented in new practice settings is somewhere between common and universal (3–5), yet program adaptation is an area with significant unresolved issues (6,7). Among those issues are differing perspectives on the value of program changes (7,8). From one perspective, they are undesirable deviations from a tried-and-true formula that lead to a voltage drop in the intervention’s efficacy (9,10). From another perspective, local tailoring of an EBI can enhance program outcomes by improving the fit of an intervention for a particular population or setting, thus allowing a process of evolution to optimize program functioning and effects (11) and fostering a sense of ownership among staff (12).

A common middle-ground solution is for intervention developers to designate certain program components as core (essential to achieving outcomes) (13), while leaving others as peripheral (nonessential or modifiable) (6,10). However, isolating the core elements that are both necessary and sufficient to achieve program effect across replications in diverse settings is often more art than science. For example, selecting core components is often based on the judgment of the program designers (10) rather than on resource-intensive systematic testing or experiential learning from replications (13), and some researchers suggest that even core program elements can be changed to beneficial effect in certain contexts (2,11).

Clearly, much remains to be learned about optimizing the EBI adaptation process. One step in this direction is to deepen our understanding of the ways that implementing organizations adapt EBIs in the real world (8). On the basis of our observations from a cross-site evaluation of the implementation of an EBI for pediatric asthma care coordination into 4 urban community settings, we propose the notion of “retrofitting” as a type of adaptation that may be common in practice but is not fully accounted for in existing theoretical frameworks. Retrofitting applies to situations in which EBIs are implemented by health and social service organizations that already offer similar services to their patients or clients. Existing services are then altered, or retrofitted, to more closely match EBI components. In this article, we present a description of retrofitting, as observed in the cross-site evaluation described, and explore the potential practical and theoretical value of this concept.

## Methods

In 2010, sites funded by the Merck Childhood Asthma Network, Inc (MCAN) to implement EBIs to address asthma disparities among children (14) were invited to submit a proposal for a second round of funding. They were asked to refine their program models to focus on evidence-based care coordination for children with asthma. Four sites received this funding: the Los Angeles Unified School District Asthma Program (Los Angeles, CA); the Children's Hospital of Philadelphia Asthma Care Navigator Program (Philadelphia, PA); the Federally Qualified Health Center-based *La Red de Asma Infantíl* de Puerto Rico (San Juan, PR); and the neighborhood-based Addressing Asthma in Englewood Project (Chicago, IL). The cross-site evaluation of the implementation process focused on the EBI Yes We Can (YWC) because it was the one EBI that all sites implemented to a significant degree. YWC is a medical-social model of care that promotes optimal clinical care while deploying community health workers to provide asthma education, link families to health and social services, and facilitate family–clinician communication (15). The goal of YWC, as described by its developers, is to “assemble a set of best practices and implement them under real-world conditions” (15), and the program has evolved over time. In a series of evaluations (all using a single-group, pre–post design), YWC is associated with many positive asthma-related outcomes, including improvements in daytime and nighttime symptoms, increased prescribing of controller medications and use of asthma action plans, reduced activity impairment, reduced school and parental work absences, and fewer emergency department visits and hospitalizations (15,16). The Centers for Disease Control and Prevention (CDC) includes a case study of YWC on its National Asthma Control Program website as a potentially effective intervention (www.cdc.gov/asthma/interventions/yes_we_can.htm).

### Identifying core components of Yes We Can

As the cross-site evaluators studying the translation of established programs into new settings, our first challenge was to determine the core components of the programs, beginning with YWC. From the literature, we determined that no published evidence clearly demonstrated which YWC components were essential to producing the outcomes. Rather, as with most studies, the outcomes were associated with the intervention as a whole. We next examined the CDC online case study of this program. This description identifies 5 “readily distinguishable” program components, but it is unclear whether these should be considered “core” components. We then conducted separate interviews with 3 YWC developers. When asked, “What about the intervention really made a difference in the outcomes?” developers emphasized different elements; for example, one focused on characteristics and actions of the community health workers, whereas another emphasized the broader concept of pairing proper medical care with attention to social aspects that can impede asthma control. On the basis of the content of the published article (15), the CDC case study, and learnings from the developers, our evaluation team used a process of consensus to form working descriptions of YWC core components (Table 1), making subjective decisions based on the apparent weight placed on each component by the various sources cited. Review of the draft core component scheme by a leading expert in pediatric asthma intervention research (Noreen M. Clark) provided additional support for its validity.

**Table 1:** Core Components of Yes We Can, Case Study of Pediatric Asthma Care Coordination, United States, 2010–2014

Component	Key Characteristics
Risk stratification: establishing levels of care	Risk based on medical severity and control, psychological risk, and social risk Care pathway and intervention activities matched to risk level

Asthma care coordinator (ACC)	Culturally and linguistically aligned with families served Provides basic education including “how to” use spacers, reduce triggers, etc Addresses social problems as they arise: working with schools, providing assistance in finding new housing, making referrals to smoking cessation programs, obtaining refills, etc Makes the family feel like a valued member of the care team

Asthma clinical care: chronic care approach	Prevention-based “asthma clinic” established with set hours to see children with asthma ACC integrated into the health care team Team members reinforce colleagues’ educational efforts Routine case conferences follow clinic hours Careful planning and integration of clinic and home visits supported by telephone calls Schedule for ongoing assessment of control Designated clinical champion

Clinical case management	Organized, systematic tracking of patients to assess needs and match services Established network for referrals

### Key informant interviews

We conducted 9 key informant interviews by telephone (average length, 80 minutes) with principal investigators (1 per site), project managers (1 per site), and field staff (asthma care coordinators; 1–3 per site). Interview questions were developed by the evaluators (M.R.J., S.C.S.) and incorporated implementation-related factors identified by Durlak and Dupre (9). The interviews included review of a YWC core components table; respondents were asked whether and how each component was implemented. Interview notes (verified and augmented with audio-recordings) were coded with both codes that we prespecified based on the theoretical constructs used to develop interview questions as well as on additional codes suggested by the interview data; “retrofitting” fell into the latter category.

## Results and Discussion

Data indicated that no site implemented all YWC components, adapted or not. One site leader described their adaptation process as follows: “We didn’t really *adapt* the EBI so much as we backed it into what we were already doing.” A leader of another site urged us to take our study of “translation” out of the evaluation, because she did not like the idea of assessing fidelity and instead saw greater value in examining the strengths and weaknesses inherent in each setting where care coordination interventions are implemented.

These comments alluded to the reality that implementing organizations often have a base of existing services and select EBI components that will 1) satisfy funder requirements for EBI implementation, 2) enhance or expand the services they already provide, or 3) both. Of the 15 core components listed in Table 1, the number that existed at project sites before funding ranged from 0 to 7 (mean, 4.8); the number added with project funding ranged from 1 to 8 (mean, 4.8); and the number that were not present at any point ranged from 2 to 10 (mean, 5.5) (Table 2). Table 3 provides examples from each site — gleaned from interviews, annual reporting forms, and conference call minutes — of how existing services were altered to better align with YWC components. For example, because children in YWC are stratified according to medical and psychosocial risk for purposes of determining intervention intensity, the school nurses in the Los Angeles Unified School District formalized a process of risk stratification based on asthma control, while continuing to informally account for psychosocial risks as they had done previously. We termed these types of changes as “retrofitting.”

**Table 2:** Implementation Level of Yes We Can Core Components at MCAN Sites, Case Study of Pediatric Asthma Care Coordination, United States, 2010–2014^a^

Core Component	Philadelphia	Los Angeles	Chicago	Puerto Rico
**Risk stratification: establishing levels of care**				
Risk based on medical severity and control, psychological risk, and social risk	3	3	2	1
Care pathway and intervention activities matched to risk level	2	3	2	1
**Asthma care coordinator**				
Culturally and linguistically aligned with families served	2	2	2	3
Provides basic education, including how to use spacers and reduce triggers	2	2	2	3
Addresses social problems as they arise, including working with schools, providing assistance in finding new housing, making referrals to smoking cessation programs, and obtaining refills	2	2	2	1
Makes the family feel like a valued member of the care team	3	2	2	3
**Asthma clinical care: chronic care approach**				
Prevention-based asthma clinic established with set hours to see children with asthma	1	2	3	1
Asthma care coordinator integrated into the health care team	3	1	1	1
Team members reinforce colleagues’ educational efforts	3	3	1	3
Routine case conferences follow clinic hours	1	1	1	1
Careful planning and integration of clinic and home visits supported by phone calls	3	1	1	1
Schedule for ongoing assessment of control	3	3	1	1
Designated clinical champion	2	2	2	3
**Clinical case management**				
Organized and systematic tracking of patients to assess needs and match services	3	3	1	1
Established network for referrals	3	2	1	1

Abbreviation: MCAN, Merck Childhood Asthma Network.

a Stage of implementation: 1 = never existed, 2 = already existed, 3 = added with MCAN funding.

**Table 3:** Retrofitting Yes We Can Core Components, Examples From 4 Program Sites, Case Study of Pediatric Asthma Care Coordination, United States, 2010–2014

Site	Yes We Can Core Component Characteristics	Existing	Retrofits
Philadelphia	ACC culturally and linguistically aligned with families, provides basic education, addresses social problems, and makes family feel like part of care team	ACCs who made home visits were already culturally and linguistically aligned with families and provided basic education	ACCs learned how to assess and address social problems and were better able to make families feel like a valuable part of the care team, because the ACCs themselves were integrated into the clinical care team

Los Angeles	Formal risk stratification process based on medical control and social and psychological risk; intervention activities matched to risk level	ACCs were registered nurses who informally accounted for risk when determining the level of care	Program implemented formal risk stratification based on asthma control; ACCs continued to informally account for social and psychological risks

Chicago	ACC integrated into the health care team	ACCs were community based; they made educational home visits but were not connected to clinical care	ACCs also recruited and provided education in clinic waiting rooms and communicated with clinical providers

Puerto Rico	Chronic care approach to asthma clinical care, including aligning educational efforts among care providers	Standard asthma care; not all clinicians using an asthma action plan	Clinical champion helped implement routine use of an asthma action plan, which reinforced colleagues’ educational efforts

Abbreviation: ACC, asthma care coordinator.

### Why the term “retrofitting,” and what are its potential benefits?

A dictionary defines “to retrofit” as “to install new or modified parts or equipment in something previously manufactured or constructed” (www.merriam-webster.com/dictionary/retrofit). For example, a homeowner might retrofit a house to make it more energy-efficient by adding insulation, or replacing windows. Similarly, program providers might retrofit existing services in a certain program area (the analog to a house) to make them more like those of an EBI. Just as it often makes more sense for homeowners to retrofit their existing house rather than build a new one, so might program providers modify what they are already doing rather than replace services to implement an EBI in its entirety. We believed that the term “retrofitting” was appropriate for the implementation-related phenomenon we sought to describe, as it denotes improving, via scientifically supported practices, something that already exists.

When EBIs are implemented in settings where services overlap with EBI elements, the 2 must be smoothly integrated. This could take the form of “tweaking” existing services to align them with EBI elements, continuing services that have the same goals as the EBI but are not in the EBI design, and basing decisions on adding EBI elements on the degree to which they would enhance, or adversely affect, current practices. Potential benefits of retrofitting include allowing organizations to keep what already works and capitalize on political support for program activities and resources needed for implementation, such as training, physical space, and time (6). Because political support and organizational integration are associated with program sustainability (17), retrofitting may enhance an organization’s ability to maintain effective innovations following the initial funding period. Finally, the notion of retrofitting elevates practice-based and local expertise, by placing value on what an organization already does.

The notion of overlapping EBI components with existing services can be found in several implementation science articles: a category from Stirman and colleagues’ program-modification typology, “Integrating the intervention into another framework — ie, selecting elements” (7); Durlak and Dupre’s (9) “Integration of new programming,” defined as “the extent to which an organization can incorporate an innovation into its existing practices and routines”; and the construct of “Organizational Fit/Compatibility,” which encompasses how well the innovation fits with existing systems, in the Consolidated Framework for Implementation Research (6). Generally, however, adaptation has been conceptualized as *changing the content or process of the EBI* to improve its fit (7), rather than *reshaping existing services* to be more similar to the EBI (ie, retrofitting).

The difference between adaptation and retrofitting may merely be one of vantage point and degree. However, we suggest that recognition of the retrofitting phenomenon can inform various stages of the EBI pipeline: the design of programs and the trials to establish their efficacy; isolating core components and preparing EBIs for dissemination; conducting needs and capacity assessments of implementing organizations; training and technical support; and evaluations of the processes and outcomes of retrofitted programs and practices. We give brief examples of how this influence might occur:

**Program design and efficacy testing:** A consideration of retrofitting may mean decreased frequency of designing programs “from scratch” (eg, in an academic setting) with increased attention to current practices and how they can be incrementally improved. Equitable community–academic partnerships may be especially valuable in this regard.

**Isolating core components:** In a retrofitting-aware approach, current practice is seen as a foundation that can be improved with evidence-based modifications. Therefore, being able to accurately identify the “active ingredients” in a program (whether processes or principles) takes on special importance. The challenges we experienced in identifying YWC’s core components have been described (18). Although solving the thorny practical and theoretical issues surrounding core components was beyond the scope of this article, it is worth considering emerging methods such as qualitative comparative analysis (19) for improving the validity of core component identification.

**Needs and capacity assessment:** EBI implementation frameworks (20,21) typically include an assessment phase — which examines an organization’s strengths, needs, and structure, among other attributes — with the purpose of informing how the EBI is subsequently adapted and implemented. One explicit aim of such a phase could be to identify where and how existing services can be retrofit with EBI components.

**Training and technical support:** Training and technical support that accommodate retrofitting would place greater emphasis on smoothly integrating EBI components into existing services and less on fidelity to the procedures used in the EBI’s original trial (5).

**Process and outcome evaluations of retrofitted programs and practices:** Miller et al (2) pose an ontological question with practical implications, “At what point does a replicated program become a new model program in its own right?” EBIs implemented via significant retrofitting, as in this study, may be considered new programs that would benefit from process and outcome evaluations. One formal approach to doing this is Rapkin et al’s Comprehensive Dynamic Trial paradigm (11). This approach prescribes careful monitoring of the performance of interventions that are implemented in new settings. This is done for purposes of quality improvement and to inform the design of embedded “mini experiments” to test the value of specific adaptations. Indeed, MCAN sites engaged in a process similar to this, as each site reported using evaluation for quality improvement (22), and 1 site (Puerto Rico) embedded a randomized experiment to compare the effects of asthma care coordination delivered in a combined clinical–home versus a clinic setting.

Several limitations apply. First, the concept of retrofitting emerged from the qualitative data collected from a single evaluation, and we did not collect data specifically addressing this concept. In future studies, instruments could be developed to measure retrofitting more explicitly. Second, retrofitting may apply to only a limited number of circumstances; for example, it may be less relevant where the innovation under study changes a single existing practice as opposed to a multicomponent package of activities and processes. Third, our sample was small, consisting of 4 sites with different patient populations, recruitment approaches, and settings. Therefore, we were not able to correlate degree of retrofitting with important implementation-related outcomes such as reach, adoption, or sustainability. Nor were we able to associate retrofitting with asthma-related outcomes among participants. Notably, each program site reported improvements in asthma-related outcomes among program participants, offering tentative evidence that the retrofits contributed to, or at least did not hinder, the success of the programs (23).

## Conclusion

We found preliminary support for the validity of the retrofitting concept, although the explanatory or practical value of this concept is yet to be determined. The key assumption underlying the notion of retrofitting is that practitioners are doing things that work and that may benefit from evidence-based modifications rather than new interventions. This perspective aligns with calls for more practice-based evidence (24) and with community-based participatory approaches to research that emphasize local knowledge, resources, and practices (25). Future research is needed to explore retrofitting in a systematic manner, using larger samples and mixed quantitative and qualitative methods to learn more about processes and effects.
